# Internistische Differenzialdiagnosen bei akuten Rückenschmerzen: Eine internistische Perspektive zu den möglichen Ursachen von akuten Rückenschmerzen

**DOI:** 10.1007/s00393-022-01257-7

**Published:** 2022-09-12

**Authors:** Nicolas F. Thalmann, Caroline Rimensberger, Manuel R. Blum, Fabian D. Liechti, Maria M. Wertli

**Affiliations:** 1 Allgemeine Innere Medizin, Universitätsspital Bern, Inselspital, Freiburgstr. 18, 3010 Bern, Schweiz; 2 Departement Innere Medizin, Kantonsspital Baden, Im Ergel 1, 5404 Baden, Schweiz; 3 Berner Institut für Hausarztmedizin (BIHAM), Universität Bern, Mittelstr. 43, 3012 Bern, Schweiz

**Keywords:** Anamnese, Klinische Untersuchung, Alarmzeichen, Internistische Grunderkrankungen, Differenzialdiagnose, Medical history, Clinical examination, Red flags, Internal underlying disease, Differential diagnosis

## Abstract

Die Mehrheit der Patienten mit akuten Rückenschmerzen weist keine schwerwiegende, zugrunde liegende Erkrankung auf. Viele internistische Erkrankungen können sich jedoch mit akuten oder chronischen Rückenschmerzen manifestieren. In der Beurteilung von Patienten mit Rückenschmerzen sind daher die Anamnese und die klinische Untersuchung wichtig, um Hinweise auf eine allfällige, zugrunde liegende Erkrankung zu erfassen. Insbesondere Alarmzeichen, die auf eine akute und lebensbedrohliche Erkrankung hinweisen, sollten dabei nicht verpasst werden. Meist ist bei fehlendem Vorliegen von entsprechenden Alarmzeichen, Risikofaktoren oder klinischen Hinweisen keine systematische Suche von internistischen Grunderkrankungen nötig. Nachfolgend sind die wichtigsten Differenzialdiagnosen und klinischen Hinweise sowie Alarmsymptome zusammengefasst.

## Abstract (en)

The majority of patients with acute back pain have no serious underlying disease; however, many internal diseases can be manifested as acute or chronic back pain. Therefore, in the assessment of patients with back pain the clinical history and clinical examination are important in order to detect indications for a possible underlying disease. Particularly red flags that indicate an acute or life-threatening disease should not be missed. In most cases where such red flags, risk factors or clinical indications are not present, no systematic search for internal underlying diseases is necessary. This article summarizes the most relevant differential diagnoses and clinical indications as well as warning symptoms.

Akute Rückenschmerzen sind ein häufiges Symptom in der klinischen Praxis. In einer kürzlich publizierten Befragung gaben 61 % der befragten Personen in Deutschland an, in den letzten 12 Monaten an Rückenschmerzen gelitten zu haben [1]. In den USA machen akute, nichttraumatische Rückenschmerzen bei Erwachsenen 2–3 % der Notfallkonsultationen aus [2], in Deutschland 2019 3,6 % der ambulanten Notfälle in der Notaufnahme [3]. Neben den degenerativen und rheumatologischen Ursachen gibt es auch viele internistische Krankheiten, die sich mit dem Symptom Rückenschmerzen präsentieren können. Diese Krankheiten können akut und lebensbedrohlich sein. Daher ist es wichtig, die Differenzialdiagnosen, klinische Hinweise und Red Flags zu kennen und zu suchen.

Bei der klinischen Beurteilung von Patienten mit akuten oder chronischen Schmerzen fließt neben der Vortestwahrscheinlichkeit für das Vorliegen einer schwerwiegenden Erkrankung die Suche nach Alarmzeichen (sog. Red Flags) ein. In der klinischen Praxis bewähren sich in erster Linie eine gezielte Anamnese der Präsentation, der Art und Intensität der Schmerzen, der Schmerzausstrahlung sowie eine gezielte klinische Untersuchung, um Alarmzeichen früh zu erkennen (Abb. 1; [4–6]). Die Wahrscheinlichkeit einer schwerwiegenden, zugrunde liegenden Erkrankung bei Patienten, die sich mit Rückenschmerzen in einer Hausarztpraxis vorstellen, liegt bei < 1 % [7]. Stellten sich Patienten mit Rückenschmerzen auf einer Notfallstation vor, lag die Prävalenz einer schwerwiegenden, zugrunde liegenden Erkrankung, die innerhalb von 30 Tagen eine nichtchirurgische oder chirurgische Intervention benötigte, bei 3–5 % [6]. Die häufigsten spezifischen Ursachen auf einer Notfallstation sind Wirbelkörperfrakturen (in 1,5 %), Malignome (0,5 %), Epiduralabszesse (0,0–0,6 %) oder vaskuläre Probleme (Ruptur eines Aortenaneurysmas/Aortendissektion in 0,6 %). In Hausarztpraxen war die Prävalenz von Wirbelkörperfrakturen 1–4 % und von Malignomen < 1 % [6, 8]. Ein Malignom ist in der Hausarztpraxis sehr selten, weswegen Smarter Medicine – Choosing Wisely und weitere Guidelines bei Patienten ohne Alarmzeichen mit akuten Rückenschmerzen keine Bildgebung empfehlen [9, 10]. Obwohl akute Rückenschmerzen ein sehr häufiges klinisches Problem sind, gibt es leider nur wenige Studien für die Validität der in der Literatur beschriebenen Alarmzeichen [5, 6]. Liegen Alarmzeichen vor, sollte eine entsprechende Diagnostik eingeleitet werden. Nachfolgend wird auf die Differenzialdiagnosen wichtiger Erkrankungen und Alarmzeichen eingegangen (Tab. 1).

**Figure 1:**
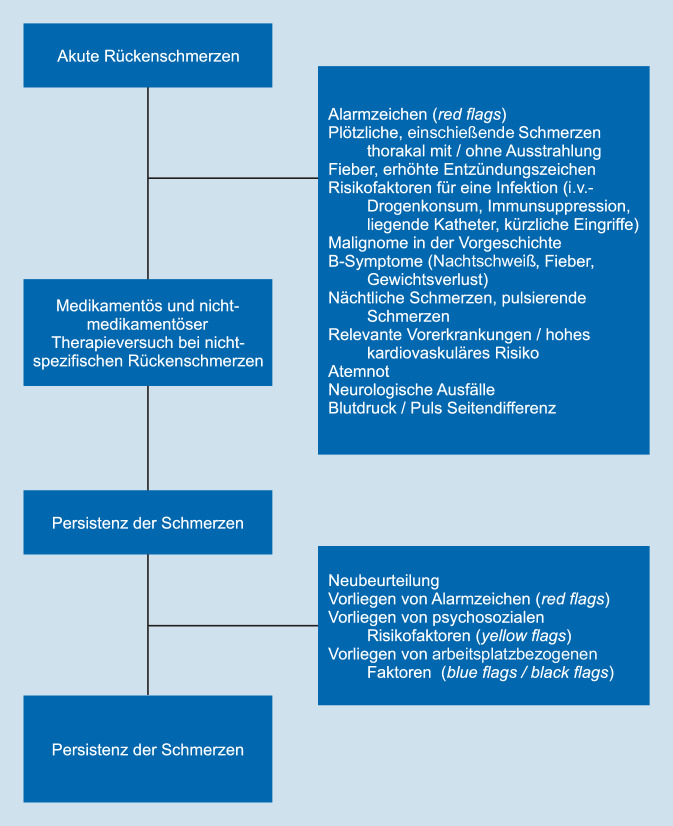

**Table 1:** 

Lokalisation	Organsystem	Differenzialdiagnose	Wichtigste Red Flags/Leitsymptome
*Thorakal*	Kardial und vaskulär	Aortendissektion	Akut einschießende, stechende, zerreißende Thorax‑/Rückenschmerzen, Blutdruck und/oder Pulsdifferenz, sensomotorische Defizite, Horner-Syndrom, Spinalis-anterior-Syndrom
		Thorakales Aortenaneurysma	Plötzlich einsetzende Abdominal‑/Thorax‑/Rückenschmerzen, Hypotonie
		ACS	Linksthorakale Schmerzen mit Ausstrahlung in linken Arm/Schulter und/oder Kiefer, Dyspnoe, Schweißausbrüche
	Pulmonal	Lungenembolie	Dyspnoe, inspiratorische Thoraxschmerzen (Pleuritis)
		Spontanpneumothorax	Ipsilaterale Thoraxschmerzen, abgeschwächtes Atemgeräusch
		Spannungspneumothorax	Thoraxschmerzen, Dyspnoe, Tachypnoe, abgeschwächtes Atemgeräusch, Hypoxämie, hypersonorer Klopfschall in der Perkussion
*Abdomen*	Vaskulär	Bauchaortenaneurysma und -ruptur	Schmerz in Bauch, Flanke oder Rücken, palpable Masse über dem mittleren Abdomen, Hypotonie/Kaltschweißigkeit bei Ruptur
		Niereninfarkt	Akute Bauch- oder Flankenschmerzen, Übelkeit, Hämaturie
	Renal	Pyelonephritis	Flanken‑, Rückenschmerzen, Fieber
		Nephrolithiasis	Akute, kolikartige Flanken- und Rückenschmerzen oft mit Ausstrahlung in die Leiste, gefolgt von Übelkeit mit Erbrechen, Hämaturie
	Gastrointestinal	Akute Pankreatitis	Gürtelförmige Bauchschmerzen, Übelkeit und Erbrechen, bei biliärer Genese acholischer Stuhl
		Pankreastumoren	Schwächegefühl, Kraftlosigkeit, Gewichtsverlust, Appetitlosigkeit, abdominelle (gürtelförmige) Schmerzen mit/ohne Ausstrahlung in den Rücken, Ikterus
		Akute Cholezystitis	Abdominelle Schmerzen im rechten, oberen Quadranten, Ausstrahlung in rechte Schulter oder Rücken, Übelkeit
		Entzündliche Darmerkrankungen	Fieber, Diarrhö, abdominelle Schmerzen
		Psoasabszess/Einblutung/Morbus Ormond	Flankenschmerzen, speziell bei Bewegung, Schmerzen im unteren Rücken
	Urogenital	Ovartorsion	Stechende Schmerzen im unteren Abdomen, in Rücken ausstrahlend, Übelkeit
*Weitere*	Malignome	Multiples Myelom	Hyperkalzämie, Anämie, skeletale Schmerzen
		Metastasen, Knochentumoren	B‑Symptome, Schmerzen, Schwäche
	Infektiös	Spondylodiszitis/Epiduralabszess	Rückenschmerzen, Fieber
		Herpes Zoster	Schmerzen entlang eines Dermatoms, Hautveränderungen
		Knochentuberkulose	Beinschwäche, Schmerzen

*ACS* akutes Koronarsyndrom

## Thorakale Differenzialdiagnosen

Bei Schmerzen im thorakalen Bereich oder ausstrahlend vom Thorax sind die wichtigsten Differenzialdiagnosen das akute koronare Syndrom, vaskuläre Ursachen (Aortendissektion, Aortenaneurysma) sowie eine akute Lungenembolie. Weitere Differenzialdiagnosen beinhalten eine Pneumonie mit pleuritischen Schmerzen, einen Pneumothorax sowie einen gastroösophagealen Reflux mit oder ohne ösophageale Spasmen. Nachfolgend werden die wichtigsten Differenzialdiagnosen und klinischen Aspekte diskutiert.

### Kardiovaskuläre Ursachen

Schmerzen insbesondere zwischen den Schulterblättern sind eine mögliche Präsentation bei einem akuten Koronarsyndrom (ACS). Frauen mit ACS berichten häufiger über Schmerzen zwischen den Schulterblättern (verglichen mit Männern Odds Ratio 2,15 [Konfidenzintervall 1,95–2,37]; [11]). Typische Symptome des ACS sind insbesondere Thoraxschmerzen (bei Männern in 89 %, Frauen 82 %), Dyspnoe (Männer 48 %, Frauen 51 %), Schweißausbrüche (Männer 38 %; Frauen 29 %) und Schmerzen im linken Arm (Männer 30 %, Frauen 25 %) [12].

Eine Aortendissektion präsentiert sich in 85 % der Fälle mit plötzlich einsetzenden Brust- und/oder Rückenschmerzen

Bei einer Aortendissektion sind plötzlich einsetzende Brust- und/oder Rückenschmerzen sehr häufig (in 85 % der Fälle) [13]. Die Schmerzqualität wird oft (in 64 %) als stechend und zerreißend beschrieben. Weitere Symptome sind neurologische Ausfälle (Paraparesen, Horner-Syndrom, Spinalis-anterior-Syndrom) oder Synkopen [14]. Ein Blutdruck- und Pulsdefizit ist in < 20 % der Fälle beschrieben. Bei einem Verlegen des Lumens der A. spinalis anterior kann es zu einer spinalen Ischämie kommen (in ca. 3 % der Fälle [15]). Dies äußert sich mit gürtelförmigen Parästhesien, Paraparesen, Störung der Blasen- und Mastdarmfunktion oder dissoziierter Sensibilitätsstörung kaudal der Läsion (d. h. Störung des Schmerz- und Temperaturempfindens bei normalem Lage‑, Vibrations- und Berührungsempfinden).

Ein thorakales Aortenaneurysma ist gewöhnlich lange asymptomatisch und kann sich bei einer Ruptur mit Rückenschmerzen präsentieren. Die Prävalenz eines asymptomatischen, thorakalen Aortenaneurysmas mit einer Größe von ≥ 5 cm lag bei 0,3 % [16], und die Wahrscheinlichkeit einer Ruptur betrug über 5 Jahre 20 % [17].

### Pulmonale Ursachen

Die wichtigsten Differenzialdiagnosen sind akute Lungenembolien (LE) sowie ein Spontanpneumothorax. Die LE präsentiert sich häufig mit pleuritischen Schmerzen (in 66 % vorliegend), die sich auch in Form von Rückenschmerzen manifestieren können. Typisch sind die inspiratorisch verstärkten Schmerzen bei einer Pleuritis. Weitere häufige Symptome sind Dyspnoe (in 73 % der Fälle), Tachypnoe (Atemfrequenz ≥ 20/min in 70 %), Tachykardie (30 %) und Husten (37 %) [18]. Pleuritische Schmerzen treten auch bei anderen Erkrankungen (z. B. Pneumonien, bei rheumatologischen Erkrankungen mit Polyserositis) auf.

Klassische Symptome des Spontanpneumothorax sind akute, ipsilaterale Thoraxschmerzen mit milder Dyspnoe [19]. Je nach Ausmaß des Pneumothorax sind ungünstige Zeichen eine reduzierte Brustwandbewegung, eine tympanitische Perkussion oder ein Fehlen des Atemgeräuschs in der Auskultation, da sie auf einen beginnenden Spannungspneumothorax – eine lebensbedrohliche Komplikation – hinweisen können [20]. Weitere Hinweise auf einen Spannungspneumothorax sind die Dyspnoe (in 38 %), Tachypnoe (in 47 % der Fälle) und Hypoxämie (in 50 %).

## Abdominelle Differenzialdiagnosen

Die wichtigsten Differenzialdiagnosen im Abdomen sind bei akuten Rückenschmerzen ein rupturiertes Aortenaneurysma, eine Pyelonephritis oder Nephrolithiasis, eine Pankreatitis oder eine akute Cholezystitis.

### Vaskuläre Ursachen

Das abdominale Aortenaneurysma ist zwar in etwa 75 % der Fälle asymptomatisch [14], wobei zunehmende Schmerzen in Bauch, Flanke oder Rücken auftreten können. Die Schmerzen strahlen oft in Rücken, Leiste oder Skrotum aus. Klinisch kann eine palpable Masse im mittleren Abdomen palpiert werden (Sensitivität stark variabel mit 29–76 %) [21]. Bei einer Ruptur des Aneurysmas treten meist starke Schmerzen in Bauch, Flanken oder Rücken auf. Bei 66 Patienten mit einem rupturierten Aortenaneurysma klagten 17 % über isolierte lumbale Schmerzen und 14 % über Schmerzen abdominell und lumbal [21]. Bei starkem Blutverlust sind eine Hypotonie oder Synkopen bis zur Schocksymptomatik möglich [14, 21].

### Renale Ursachen

Die klassischen Symptome einer Pyelonephritis beinhalten Flankenschmerzen (in 48 % der Fälle), Fieber (in 44 %) und Übelkeit und Erbrechen (in 24 %) [22]. Stärkste, akute Flanken- (57 %) oder Rückenschmerzen (43 %) sind zudem Leitsymptome einer Nephrolithiasis [23]. Weitere Begleitsymptome sind Übelkeit mit Erbrechen in 74 % und Hämaturie in 68 %. Nierensteine sind jedoch viel häufiger asymptomatisch und ein Zufallsbefund bei einer Sonographie (Prävalenz bei Ultraschalluntersuchungen zwischen 30 und 46 %) [24].Ein Niereninfarkt kann sich mit akuten Flankenschmerzen (in 49 %) und Bauchschmerzen (51 %) manifestieren [25]. Weitere häufig genannte Symptome sind Übelkeit (28 %), Erbrechen (20 %) oder Fieber (20 %).

### Gastrointestinale und urogenitale Ursachen

Eine akute Pankreatitis präsentiert sich am häufigsten mit Abdominalschmerzen, die gürtelförmig in den Rücken ausstrahlen (90 %) [26]. Weitere Symptome sind Erbrechen (in 80 %), Fieber (60 %) sowie ein paralytischer (Sub‑)Ileus (70 %). Differenzialdiagnostisch ebenfalls mit Rückenschmerzen können sich Pankreastumoren manifestieren. Bei Patienten mit exokrinen Pankreastumoren gaben 49 % der Patienten Rückenschmerzen an [27]. Weitere häufige Symptome waren Schwächegefühl und Kraftlosigkeit (86 %), Gewichtsverlust (85 %), Appetitlosigkeit (83 %) und Bauchschmerzen (79 %).

Eine akute Cholezystitis kann sich mit Schmerzausstrahlung in die rechte Schulter oder im Rücken präsentieren [28]. Typischerweise bestehen Abdominalschmerzen im oberen rechten Quadranten (Sensitivität 81 %) und eine lokale Druckdolenz (Sensitivität 77 %). Die höchste Spezifität zeigte sich bei einem positiven Murphy-Zeichen mit 87 % [28].

Seltene Ursachen für Rückenschmerzen sind ein peptisches Ulkus [29], Pathologien im M. psoas [30, 31] und eine Ovartorsion [32]. Liegt ein Psoasabszess vor, manifestiert sich dies in ca. 50 % der Fälle mit bewegungsabhängigen Flankenschmerzen [30]. Ebenfalls mit Flankenschmerzen können sich Einblutungen (bei Antikoagulation) oder eine retroperitoneale Fibrose (Morbus Ormond) manifestieren. Bei der retroperitonealen Fibrose beklagten 60 % Schmerzen im unteren Rücken, 57 % im Abdomen und/oder 53 % in den Flanken mit Ausstrahlung in die Hüftregion (30 %) oder in die Leiste (24 %) [31].

Bei einer Ovartorsion treten fast immer Schmerzen im unteren Abdomen auf, davon in 51 % der Fälle mit Ausstrahlung in Flanke, Rücken oder Leiste [32].

## Weitere Differenzialdiagnosen

Auch systemische Erkrankungen können sich mit Rückenschmerzen manifestieren. Dazu gehören Malignome (primär und metastasiert) sowie neuropathische Schmerzen (z. B. bei einem Herpes Zoster).

### Maligne Ursachen

Ein multiples Myelom präsentiert sich häufig (in 58 %) mit skeletalen Schmerzen. Weitere Symptome beinhalten Anämie (in 73 %), Niereninsuffizienz (48 %) sowie Müdigkeit und Schwäche (32 %) [33].

Die häufigsten Malignome, die eine Rückenmarkkompression verursachen, sind Metastasen von Lungenkarzinomen (25 %), Prostatakarzinomen (16 %), multiplen Myelomen (11 %) sowie Hodgkin- und Non-Hodgkin-Lymphomen (6–8 %) [34]. Metastasen sind am häufigsten thorakal (60 %) sowie lumbal (30 %) und nur in 10 % zervikal lokalisiert [2].

### Infektiöse Ursachen

Die wichtigste infektiöse Differenzialdiagnose ist der Epiduralabszess, der aufgrund eines potenziell lokal schnell fortschreitenden Geschehens zur Myelonkompression führen kann. Die Symptome eines Epiduralabszesses sind oft unspezifisch. Lediglich 10 % der Patienten präsentieren sich mit der typischen Trias aus Fieber, Rückenschmerzen und neurologischen Defiziten [2]. Rückenschmerzen sind das häufigste Symptom und liegen bei 70–90 % der Patienten vor. Nur 66 % der Patienten haben Fieber, während 26 % über Muskelschwäche, 24 % über Inkontinenz und 13 % über Sensibilitätsstörungen klagen. Die Ursache ist in der Hälfte der Fälle eine hämatogene Dissemination [35]. Dabei können Infektionen, ausgehend von der Haut, Weichteilen, den Harnwegen, oder eine Endokarditis der Infektionsherd sein. In einem Drittel der Fälle breitet sich die Infektion per continuitatem bei infiziertem Nachbargewebe aus. Des Weiteren bestehen iatrogene Risikofaktoren wie eine Infiltration oder Operationen [36]. Anderweitige bakterielle Ursachen beinhalten die Spondylodiszitis und paraspinale Abszesse. In einer Studie mit mikrobiell nachgewiesenen Spondylodiszitiden präsentierten sich 97 % der Patienten mit Rücken- oder Nackenschmerzen, 97 % mit einer Berührungsempfindlichkeit und 94 % mit einer Bewegungseinschränkung im Rücken.

Die wichtigste infektiöse Differenzialdiagnose ist der Epiduralabszess

Neuropathische Schmerzen entlang eines Dermatoms sind die typische Präsentation eines Herpes Zoster. Die Schmerzen sind oft brennend, einschießend oder stechend. In einer Studie gaben in der Prodromalphase 70–80 % Schmerzen in jenen Dermatomen an, in denen im weiteren Verlauf typische Hautveränderungen (erythematöses, makulöses Exanthem gefolgt von einer papulösen Phase mit Vesikeln) auftraten [37].

Die Tuberkulose (in der Regel eine Reaktivierung) ist ein medizinisches Chamäleon und kann sich sowohl im Knochen als auch in den Weichteilen paravertebral manifestieren. Die Knochentuberkulose ist am häufigsten thorakal (56 %) und in 23 % lumbal lokalisiert [38]. Je nach Lokalisation und Ausdehnung können eine Beinschwäche (70 %), eine Deformität (46 %) und Schmerzen (21 %) klinische Zeichen sein. Weitere Symptome sind oft unspezifisch wie Gewichtverlust, Nachtschweiß oder eine Systementzündung.

## Fazit für die Praxis

- Präsentieren sich Patienten mit akuten Rückenschmerzen, sollen in einer detaillierten Anamnese und sorgfältigen klinischen Untersuchung Hinweise auf Alarmsymptome und Hinweise auf das Vorliegen von zugrunde liegenden Erkrankungen gesucht werden.
- Aufgrund der hohen Spontanheilungsrate ist in den meisten Fällen, wenn Alarmzeichen fehlen, keine weiterführende Abklärung notwendig.
- Wichtig ist jedoch, dass bei Persistenz der Beschwerden eine sorgfältige Reevaluation erfolgt.
